# Supplementary material for: Cortical processing for the vestibular and visual input of egomotion in macaque monkeys: Separate networks with targeted convergence
Source: Imaging Neurosci (Camb). 2026 Jan 12;4:IMAG.a.1093. doi: 10.1162/IMAG.a.1093 (PMC12797147; doi:10.1162/IMAG.a.1093)
Supplement: Supplementary Material [file IMAG.a.1093_supp.pdf]

Supplementary Material

Cortical Processing for the Vestibular and Visual Input of Egomotion in Macaque Monkeys: Separate Networks with Targeted Convergence

Sarah Marchand[1]🖂, Vanessa De Castro[1], Elisabeth Excoffier[1], Marie-Alphée Laurent[1], Maxime Rosito[1], Nathalie Vayssière[1], Benoit R. Cottereau[1], Alexandra Séverac Cauquil[1], Jean-Baptiste Durand[1]🖂

[1] Centre de Recherche Cerveau et Cognition, Université de Toulouse, CNRS, Toulouse, France

🖂 Corresponding authors
Sarah Marchand - sarah.marchand@univ-tlse3.fr
Jean-Baptiste Durand - jbdurand@cnrs.fr

Summary

***Supplementary Figure 1*……………………………………………………………. 2**

Cortical activations evoked by GVS at the group level in CHARM regions S1/2, S3a/b and M1.

***Supplementary Figure 2*……………………………………………………………. 3**

ROI analyses of the vestibular and visual activations with respect to the CHARM atlas.

***Supplementary Figure 3*……………………………………………………………. 4**

Zoomed-in views of visuo-vestibular activations in key dorsal cortical regions.

***Supplementary Figure 4*……………………………………………………………. 5**

Comparison between known vestibular regions and activations observed in the present study.

Supplementary Figure 1.

Cortical activations evoked by GVS at the group level in CHARM regions S1/2, S3a/b and M1.

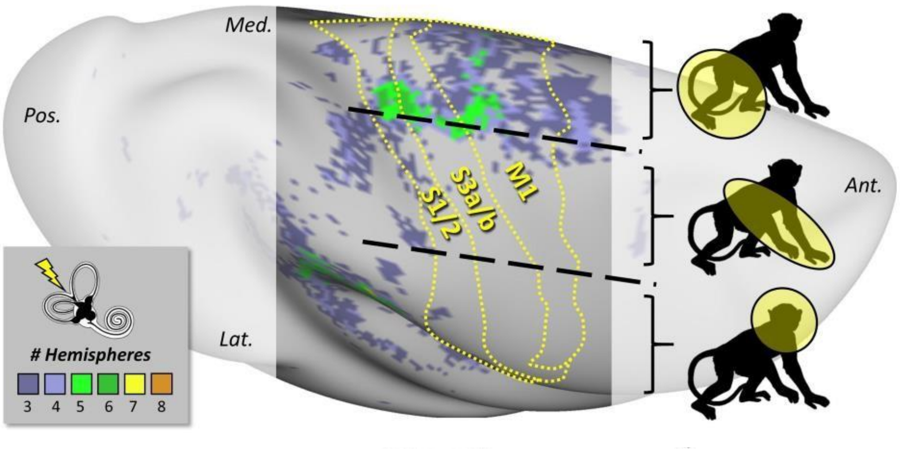

Yellow dashed lines represent the borders of areas S1/2, S3a/b and M1 as defined by the CHARM atlas. Somatotopic divisions are shown in black dashed lines and their corresponding somatotopic areas are shown on the monkey figures to the right: the medio-dorsal third is dedicated to the feet and hindlimbs, the central third represents the trunk and forelimbs and the latero-ventral third is related to the face. Medial (Med.), lateral (Lat.), anterior (Ant.), and posterior (Pos.) directions of the inflated right hemisphere are indicated.

Supplementary Figure 2.

ROI analyses of the vestibular and visual activations with respect to the CHARM atlas.

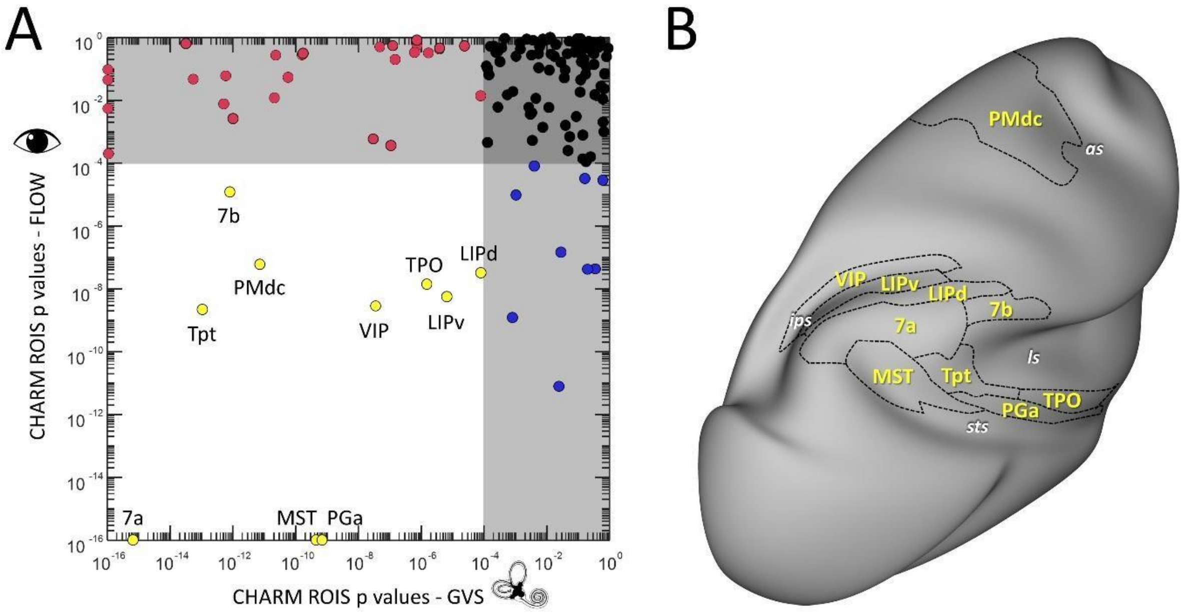

(A) *For each area of the CHARM atlas (n=135) and each monkey, we calculated the p-value for the vestibular ("GVS ON" > "GVS OFF") and visual ("1P" > "9P") contrasts (see Methods section). For each modality, individual p-values were combined using Fisher's combined probability method. Combined p-values for GVS and FLOW are shown for the 135 CHARM areas (circular symbols). Black, magenta, blue and yellow symbols stand for areas statistically non-significant for both GVS and FLOW, significant for GVS only, significant for FLOW only and significant for both GVS and FLOW, respectively (p < 0.05 after Bonferroni correction for the number of CHARM areas).* ***(B)*** *The borders of the CHARM areas significant to both GVS and FLOW are shown on an inflated representation of the right hemisphere of the NMT template.*

As a complement, we used the CHARM atlas to perform a more conventional ROI analysis, computing the mean BOLD PSC for GVS and optic flow for each of the atlas regions and in each individual. pvalues obtained in the four individuals (eight hemispheres) were combined using Fisher's combined probability method and corrected for the number of CHARM regions (Bonferroni correction, pFWE < 0.05). The results of this ROI analysis are shown in **Supplementary Figure 2**. Twenty-four areas showed statistically significant responses to GVS alone (red symbols), nine areas responded significantly to optic flow alone (blue symbols), and importantly, ten areas demonstrated significant bimodal responses (yellow symbols, $p < 0.05$ after Bonferroni correction). These regions comprise the areas previously mentioned in the other analyses: parietal area 7 (7a/7b), Tpt (VPS), PMdc (FEFsem), MST (MSTd), LIP (LIPd/LIPv) and VIP. In addition, this statistically more powerful approach reveals two additional areas, the temporo-paerieto-occipital area (TPO) and PGa, both lying in the upper bank of the sts, and thought to be parts of the superior temporal polysensory (STP) area (Bruce *et al.*, 1981; Seltzer & Pandya, 1994).

Supplementary Figure 3.

Zoomed-in views of visuo-vestibular activations in key dorsal cortical regions.

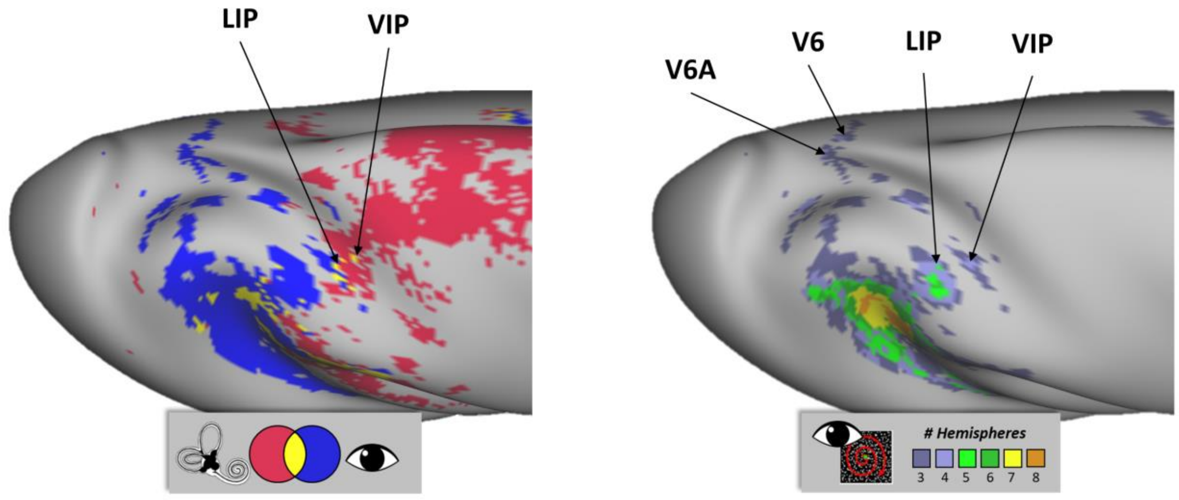

Dorsal views of the cortical maps from ***Figures 4B*** *and* ***4C*** *are shown with a focus on VIP, LIP, V6, V6A, highlighting the activations in these areas. Activations are color-coded as in the main figures. This zoomed-in presentation facilitates the visualization of the spatial relationship between visual and vestibular responses, complementing the full maps in Figure 4. Somatosensory regions S1/2/3 are not shown here, as they are already presented in a zoomed-in format in* ***Supplementary Figure 1****.*

Supplementary Figure 4.

Comparison between known vestibular regions and activations observed in the present study.

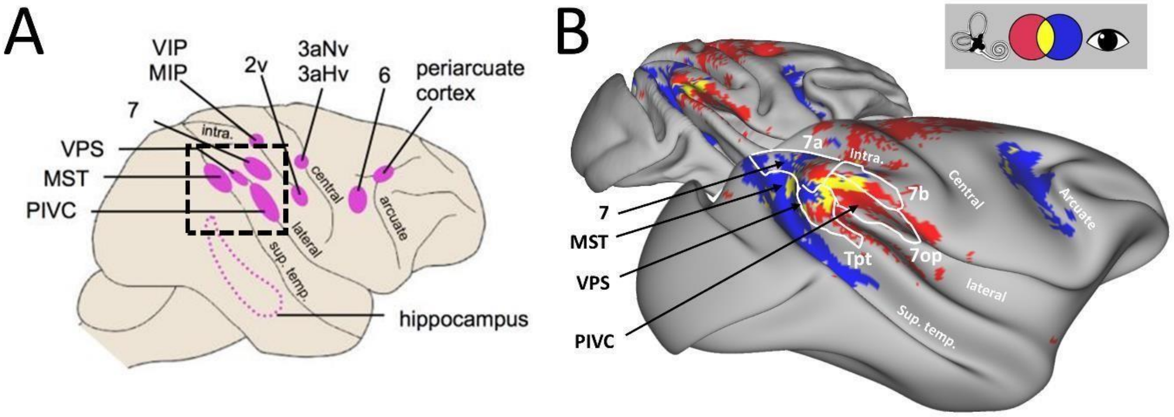

(A) *Schematic representation of cortical vestibular areas identified in macaque monkeys (from Lopez & Blanke, 2011), including regions along the intraparietal, central, and lateral sulci, as well as the peri-arcuate cortex.* ***(B)*** *Cortical activations elicited by vestibular (red) and visual (blue) self-motion signals, with their overlap shown in yellow. The main sulci are indicated for reference (intra-, central, arcuate, lateral, and superior temporal sulci).*
